# Editorial: The Social-Ecological Context of Health Literacy

**DOI:** 10.3389/fpubh.2022.897717

**Published:** 2022-04-26

**Authors:** Kevin Dadaczynski, Susie Sykes, Éva Bíró, Karolina Kósa

**Affiliations:** ^1^Department of Nursing and Health Science, Fulda University of Applied Sciences, Fulda, Germany; ^2^Centre for Applied Health Science, Leuphana University of Lueneburg, Lueneburg, Germany; ^3^Institute of Health and Social Care, London South Bank University, London, United Kingdom; ^4^Department of Public Health and Epidemiology, Faculty of Medicine, University of Debrecen, Debrecen, Hungary; ^5^Department of Behavioural Sciences, Faculty of Medicine, University of Debrecen, Debrecen, Hungary

**Keywords:** social-ecological system, health literacy, determinants of health, editorial, complex intervention

## Introduction

Most recent empirical findings from the WHO European Region indicate a limited ability to find, understand, critically assess and apply health-related information for between 25% (Slovenia) and 72% (Germany) of the adult population (1). Moreover, it has been widely shown that limited health literacy is associated with poor health behavior, lower use of health screenings, more hospitalization and lower general health (2, 3). With regard to economic effects, limited health literacy causes additional costs that range from 3 to 5% of the annual total health care costs (4). Given these findings, it is not surprising that health literacy is high on the public health agenda with 19 Member States of the WHO European Region having a health literacy policy on a national or local level (5).

Although conceptionalized as a dual relation between individual skills and the complexity of the system in which health related information is provided (6), health literacy has long been focused on individual capabilities, consequently neglecting the role of the system. The reasons are manifold and include, amongst others, limited knowledge about the interaction of different health literacy dimensions, but also because of a hesitancy toward complex intervention approaches and their evaluation. However, as emphasized by Sentell et al. (7), humans are social beings whose skills and actions are constantly shaped by social and environmental factors. The infodemic, that is, the rapid spread of vast numbers of reliable and unreliable information accompanying the COVID-19 pandemic might serve as a current example (8). Limited health literacy in pandemic times is compounded by the increasing complexity of digital information infrastructures which may lead to information overload, and the difficulty of deciding which information (source) is trustworthy. This exceeds the individual responsibility and requires greater accountability by media providers to create information environments that are not only relevant but also easy to navigate and understand (9).

Against this background, the current Research Topic aims to explore the concept of health literacy within a social-ecological framework of health and build understanding of how it can be developed beyond an individual level at organizational, community, and population levels.

## Health Literacy within a Social-Ecological Framework

Social-ecological frameworks of health have several predecessors in various disciplines. Emile Durkheim's concept of society as a level of reality above and beyond the biological level is an early example of thinking in systems (10). Known as the founding father of the General System Theory, von Bertalanffy (11) stressed the need to explain complex phenomena by considering the systems in which they occur and to study them as a whole, including not only their parts but their interactions within and without. This has been taken up and further developed by Uri Bronfenbrenner with his ecological system theory. With the aim of developing a model for child development, Bronfenbrenner assumed that human development takes place in a complex ecological environment which he conceived as “a set of nested structures, each inside the next, like a set of Russian dolls” [(12), p. 3]. While his theory initially included five systems (micro-, meso-, exo-, macro- and chronosystems), Bronfenbrenner later emphasized the relevance of biological and genetic aspects of human development.

These developments have had a significant impact on public health research and one of the most prominent examples is the rainbow model of health determinants of Dahlgren and Whitehead (13). Through a series of layers, this model visualizes the major interconnected domains of factors impacting on population health. Below the overarching societal environment (e.g., political, socioeconomic and cultural conditions), living and working conditions are posited such as education, housing or unemployment. Another level includes social factors influencing health such as social support from friends, family and the neighborhood, while behavioral actions (e.g., physical activity, nutrition) are summarized as individual lifestyle factors. Although widely used, there are only a few examples embedding health literacy in a social-ecological context. In their recent article, Schulenkorf et al. (14) report the results of an interview study with experts about their definition of child and adolescent health literacy. Using Bronfenbrenner's socio-ecological model, aspects of personal health literacy were mentioned most often while factors related to the organizational environment were mentioned the least. Another example comes from Rowland et al. (5) who developed a Health Literacy Policy Model to analyze health literacy policies in the WHO European Region on four societal levels (system, organization, communities, and individuals) along six vectors (e.g., education, lived environment, employment, media, digital health, health services).

## Summary of the Articles

This Research Topic comprises 14 articles, most coming from Europe (e.g., Hungary, Germany, Portugal), followed by Asia (Afghanistan, China) and North America. They draw on a range of empirical methods including quantitative (*n* = 9), qualitative methods (*n* = 1), mixed methods (including a review and qualitative data), and three concept articles.

Applying the rainbow model by Dahlgren and Whitehead (13) most articles (*n* = 9) address the individual level exclusively or with some links to other layers. For example, Schneider et al. report a first attempt to measure mental health literacy among adults from Zurich/Switzerland. Results indicate a low mental health literacy for almost half of the respondents. In another study Chawłowska et al. explore reproductive health literacy and fertility awareness among Polish female students and report highest knowledge scores for older and medical university students. Gender and age-specific studies come from Afghanistan (Harsch et al.) and Hungary (Papp-Zipernovszky et al.), while a study by Tsakpounidou et al. shows low levels of stroke-related knowledge amongst pre-school aged children. Tang et al. focus on two aspects of health literacy, that is, information seeking and evaluation among African American individuals.

Some of these studies link the individual level with some aspects of the living and working environment. These mostly include educational and socio-economic aspects such as the study by Harsch et al. which reveals education as a significant predictor of low health literacy in women from Afghanistan. Gomes da Silva et al. confirm the important role of the educational status for COVID-19 related health knowledge among Portuguese adults. Carl et al. take a more general perspective regarding the relevance of the environment for the physical activity–related health competence (PAHCO) model and extract three potential solutions for the relationship between competence and environment.

With regard to the community, Li et al. observe substantial geographic variation in health literacy in their population-based study covering 25 provinces of China. Educational level and socioeconomic status are significantly associated with health literacy, and these relations vary across the regions. In turn, Bíró et al. report no relationship in health literacy by place of residence (capital, urban, rural) but educational attainment and social support prove to be significant determinants of health literacy with some variations between different types of settlement. Thus, this study addresses the social network level of the rainbow model. Dadaczynski et al. focus more directly on the community and school level by introducing a fully tailored-based gamified intervention framework that aims at strengthening navigation health literacy. As emphasized by Dahlgren and Whitehead (13), unemployment and health care reflect living and working conditions that impact health. Both determinants are addressed by Samkange-Zeeb et al. and Szabó et al. While the first group collate evidence on health literacy among unemployed people through triangulating interviews and scoping review data, the latter measure the comprehension of available patient educational materials among different user groups.

Last but not least, one article address the wider political context shown in the outermost layer of the Dahlgren-Whitehead model. In their concept analysis, Schulenkorf et al. link the mandatory curriculum on media literacy with dimensions of health literacy. Following their line of argument, health literacy could be more easily implemented in schools if aligned systematically with the curriculum and instruction on media and digital literacy.

## Author Contributions

KD wrote the first draft of the manuscript. SS, ÉB, and KK revised the manuscript. All authors contributed to the manuscript and approved the submitted version.

## Conflict of Interest

The authors declare that the research was conducted in the absence of any commercial or financial relationships that could be construed as a potential conflict of interest.

## Publisher's Note

All claims expressed in this article are solely those of the authors and do not necessarily represent those of their affiliated organizations, or those of the publisher, the editors and the reviewers. Any product that may be evaluated in this article, or claim that may be made by its manufacturer, is not guaranteed or endorsed by the publisher.
